# Effect of Freeze-Thaw Cycles on Triaxial Strength Property Damage to Cement Improved Aeolian Sand (CIAS)

**DOI:** 10.3390/ma12172801

**Published:** 2019-08-30

**Authors:** Jun Li, Fengchi Wang, Fu Yi, Fengyuan Wu, Jiashun Liu, Zhenhuan Lin

**Affiliations:** 1 School of Civil Engineering, Shenyang Jianzhu University, Shenyang 110168, China; 2 School of Transportation Engineering, Shenyang Jianzhu University, Shenyang 110168, China; 3 College of Architecture and Transportation, Liaoning Technical University, Fuxin 123000, China; 4 College of Civil Engineering, Liaoning Technical University, Fuxin 123000, China

**Keywords:** cement improved aeolian sand, freeze-thaw damage, digital image process, fractal dimension, CT scanning

## Abstract

Natural aeolian sand has the characteristics of low cohesion and poor water stability. In order to improve its crack resistance properties in the process of freeze-thaw cycles, P.O 42.5 ordinary Portland cement was added to form a mixture called cement improved aeolian sand (CIAS). SEM was used to analyze the microscopic micro-structure of CIAS at different times (7 days and 28 days). The mechanical properties of CIAS samples affected by freeze-thaw cycles were tested in a triaxial instrument, and gray-scale images of the three-phase distribution in the CIAS after freeze-thaw cycling were obtained by computed tomography (CT) scanning technology. The pore characteristic parameters (pore area, fractal dimension, and crack length) were studied by digital image process technique. Based on classical Griffith fracture theory, the development of the crack length and crack width with increasing freeze-thaw cycles is determined. Assuming that the pore area subordinates to the Weibull distribution, the parameters of the Weibull distribution, the damage evolution defined by the elastic modulus attenuation, and the pore area development of CIAS were determined. Research shows the cohesion decreases and internal friction angle increases with increasing cycle numbers. Three development patterns are observed: crack growth, crack closure, and crack merging, and the three patterns interact during freeze-thaw cycling. Furthermore, the fractal dimension of the pore edge fluctuates with the increasing number of freeze-thaw cycles. This work provides a theoretical basis for the application of aeolian sand and develops a method for disaster prevention in applications of freeze-thaw cycling.

## 1. Introduction

Aeolian sand is a natural granular medium that is universally distributed in the northwestern and northeastern regions of China, such as the Shanxi province and inner Mongolia. This particular material originates from the weathering of rocks and is formed by wind transport. If aeolian sand could be applied to pavement base filling used in engineering applications, it could not only alleviate the shortage of building materials but also solidify the aeolian sand and reduce sand blowing due to weather. The other point of concern is water migration during freeze-thaw cycles, which accounts for the phenomenon of alternating frost heaving and thawing settlement and structure cracking [1,2]. Moreover, because changes in both the load and temperature are involved, especially the change of season from winter to spring, complex physico-chemical processes occur inside the soil mass and may further lead to damage of the soil structure [3].

The mechanical properties of soils after freeze-thaw cycling are directly related to the engineering performance of geotechnical structures such as the bearing capacity of foundations and the sliding resistance force in artificial and natural slopes [4,5,6]. Therefore, freeze-thaw stability is crucial for aeolian sand engineering, and the mechanical properties of this particular material can be improved by the hydration reaction of cement; this can be called cement improved aeolian sand (CIAS) and can indirectly be used as the filling material of foundations, thereby solving some engineering problems, such as mud boiling and pavement cracking [7,8]. CIAS is an environmentally friendly road base construction material that meets the stringent requirements of engineering stability in seasonal frozen areas and promotes environmentally friendly construction practices. Therefore, it is necessary and significant to study the stability of the mechanical properties of CIAS in freeze-thaw cycles.

Large numbers of experimental results have shown that the shear strength of soils generally decreases after freeze-thaw cycling, the degree of which depends on external factors such as moisture content [9] and dry density values [10]. Thus, the particular character of soil should also be concerned with, e.g., the surcharge loads [11] and fiber volume fractions [12]. To gain a further understanding of the effect of freeze-thaw weathering on the damage to soil or rock, many scholars have analyzed the freeze-thaw induced damage from the perspective of fracture energy [13,14,15,16]. In recent years, the use of fly ash, hydrated lime, and other reactive powders in sand has been widely reported throughout the literature. The potential for obtaining enhanced mechanical properties of such blends seems promising [17,18]. Computed tomography (CT) scanning and ultrasonic inspection have grown increasingly popular in freeze-thaw damage monitoring applications [16,19,20]. 

The above-mentioned works emphasize the strength damage behaviors of soils and concretes without considering the whole evolutionary process of pores and cracks within the materials. As cracks grow and merge, the reduction of both the resilience modulus and peak strength has frequently been discussed by scholars [21,22], and these phenomena arise from the fact that most of the engineering problems associated with roadbed disasters occur in seasonally frozen zones. Based on fact that the addition of siliceous fly ashes into the concrete can improve its fracture toughness and decrease the crack width [23,24], to analyze the evolutionary characteristics of pores and fractures, CT scanning and IPP (Image-Pro Plus) image processing technology can be used to analyze the damage evolution laws of CIAS after freeze-thaw cycling. This paper derives a model for the evolution of CIAS damage and proposes a method for performing microscopic fracture analysis on the freeze-thaw damage of CIAS structures based on mechanical testing results and CT scanning. This study is expected to offer a basis for disaster prevention and the control of freeze-thaw-resistant CIAS road base in the future.

## 2. Test Program

### 2.1. Materials

**Figure 1:**
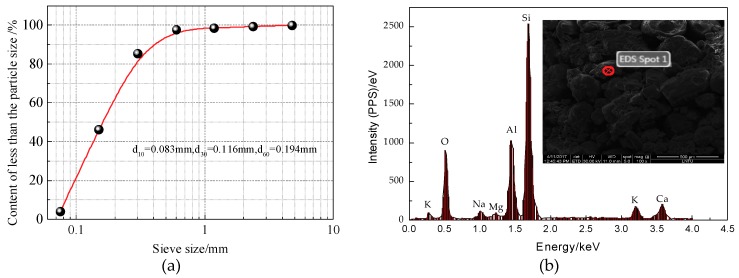
Grading curve of aeolian sand: (**a**) particle gradation; (**b**) EDS Energy Spectrum Analysis.

The soil samples were taken from an embankment filled with aeolian sand in the TJ-10 section of the Beijing-Shenyang passenger dedicated company in Fuxin, Liaoning province, China. The basic physical and mechanical parameters of the samples are listed in Table 1, and the chemical composition of aeolian sand samples listed in Table 2. The particle gradation and chemical composition and the chemical element analyzed by EDS (Energy Dispersive X-Ray Spectroscopy) are shown in Figure 1.

As seen in Table 1, the cohesion strength of natural aeolian sand is quite low, and the shear strength is mainly determined by the self-locking and embedding between particles. Once aeolian sand encounters water, the effective contact stress between particles is rapidly reduced, and the shear strength is significantly weakened. The non-uniformity coefficient $C_{u}=2.34$, the curvature coefficient $C_{c}=0.834$, and the mass of particles smaller than 0.075 mm exceeds 97.58% of the total mass; therefore, the aeolian sand is a discontinuous soil with a uniform gradation. As seen from Table 2, aeolian sand is mainly composed of SiO_2_ and CaO, which account for more than 80% of the total mass.

### 2.2. Preparation

To prepare the CIAS samples, the weight of distilled water required was calculated based on the target moisture content. Here, the target moisture content was controlled to a value of 21.2%. After sealing the CIAS samples for 24 h and maintaining the samples in a stationary environment, P.O 42.5 ordinary Portland cement was added to the wet soil according to the water-cement ratio required by each particular experiment. The initial setting time was 48 min, and the final setting time was 10 h; thus, it was important to ensure that the mixing time did not exceed the initial setting time of the cement [25].

**Figure 2:**
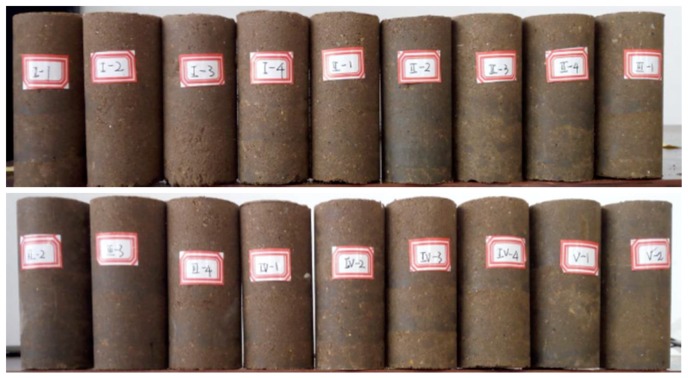
Prepared cement improved aeolian sand (CIAS) sample.

The samples were prepared and compacted using a GDS three-lobe saturator of Shenyang jianzhu University (manufactured by GDS, UK) according to the target of 95% compactness, and then, the contact surface was roughened in the five layers to ensure good contact. Cylindrical specimens with a diameter of 39.1 mm and height of 80 mm were fabricated (Figure 2). The samples were wrapped with preservative film and maintained at room temperature in the curing box to ensure full contact between the cement hydration products and sand particles. The preparation time for these testing materials was 7 days, with the freeze-thaw cycle tests beginning on the 8th day.

**Figure 3:**
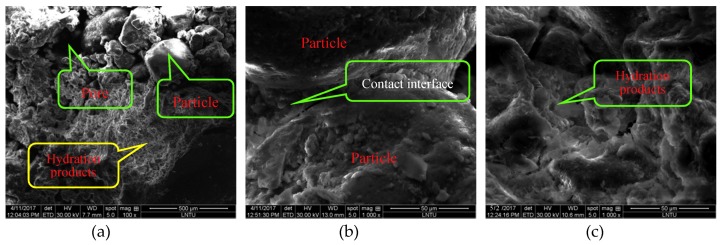
SEM micrograph of CIAS improved with the addition of 5% cement content, observed as the presence of hydration products between sand grains and the in-filling of pores. (**a**) CIAS Mag. ×100; (**b**) 7 days Mag. ×1000; (**c**) 28 days Mag. ×1000.

Based on the experimental results of ref. [26], the addition of 5% cement content to the CIAS samples is sufficient to ensure their stability. The microstructure of the CIAS with 5% cement content was observed by Quanta 250 SEM (Scanning Electron Microscope, Thermo Fisher Scientific, Waltham, MA, USA) produced in the US, as shown at 100× magnification, 1000× magnification at 7 days and 28 days in Figure 3, respectively.

As shown in Figure 3, it can be seen that the cements between aeolian sand particles in 7 days are very scarce and are in the initial stage of hydration, while those in 28 days are heavily packed among grains and are in the stable stage of hydration. The aeolian sand was greatly improved by the addition of 5% cement, which effectively filled the pores. Therefore, from a mesoscopic point of view, the cementation improved the material properties by enhancing the cohesion between the aeolian sand particles. The cement hydration reactants connect the loose aeolian sand particles, forming a complete soil skeleton. In addition, the hydration products fill the voids between the aeolian sand products, compacting its spatial structure.

### 2.3. Experimental Design

Consolidated undrained (CU) shear tests of CIAS were carried out after freeze-thaw cycling under the confining pressures of 50, 100, and 200 kPa. The freezing process was completed in a freeze-thaw cycle refrigerator, and the melting process was carried out in a thermostat. Freezing was accomplished by holding the samples at −10 °C for 12 h, and the melting process was carried out at room temperature (25 °C) for 12 h, thus completing one freeze-thaw cycle. Various numbers (0, 1, 3, 6, 10, and 15) of freeze-thaw cycles were tested. Based on the results of this study, 8–12 freeze-thaw cycles is sufficient for understanding the effect of freeze-thaw cycles on strength [27]. A GDS permafrost triaxial apparatus was selected for consolidated undrained (CU) testing, and strain control mode was adopted for all tests. The loading rate was held at 0.5 mm/min, and the control condition was the occurrence of peak stress or when vertical strain reached 10%. Under the confining pressure, the specimen was fully consolidated, and then deviatoric stress was developed by applying an axial stress. The test data were collected automatically using the data acquisition system paired with the equipment.

## 3. Test Results and Analysis

### 3.1. Effect of Freeze-Thaw Cycles on Physical Properties

To study the effects of freeze-thaw cycles on the physical parameters (height, volume, and mass) of CIAS before and after freeze-thaw cycling, several physical experiments were carried out, and the measured physical parameters listed in Table 3.

As seen in Table 3, with the increase of the number of freeze-thaw cycles, the height and volume of the specimens gradually increased. When the number of freeze-thaw cycles exceeded three, the height and volume of the specimens remained relatively stable; this indicated that the internal properties of the samples have been greatly adjusted, allowing the samples to achieve a new equilibrium. During the freezing process, a temperature gradient occurs across the inside and outside of the sample. Due to the closed nature of the system during the freezing state, the moisture in the CIAS samples gradually migrates to the cold peak surface outside of the sample, at which point ice crystals precipitate on the surface of the sample. The superficial water content of the CIAS samples reaches a saturated state due to moisture migration. Due to the subfreezing temperature, the frost heaving force first develops near the outer surface, which destroys the cementation between the particles within the sample. When the temperature rises, the ice crystals near the outer surface first melt and then flow out of the pore, resulting in a reduced mass. From the mesoscopic point of view, freeze-thaw cycles can cause irreversible expansion of the voids in the samples, thereby damaging the strength and elastic characteristics of the CIAS.

### 3.2. Effect of Freeze-Thaw Cycles on Shear Strength

**Figure 4:**
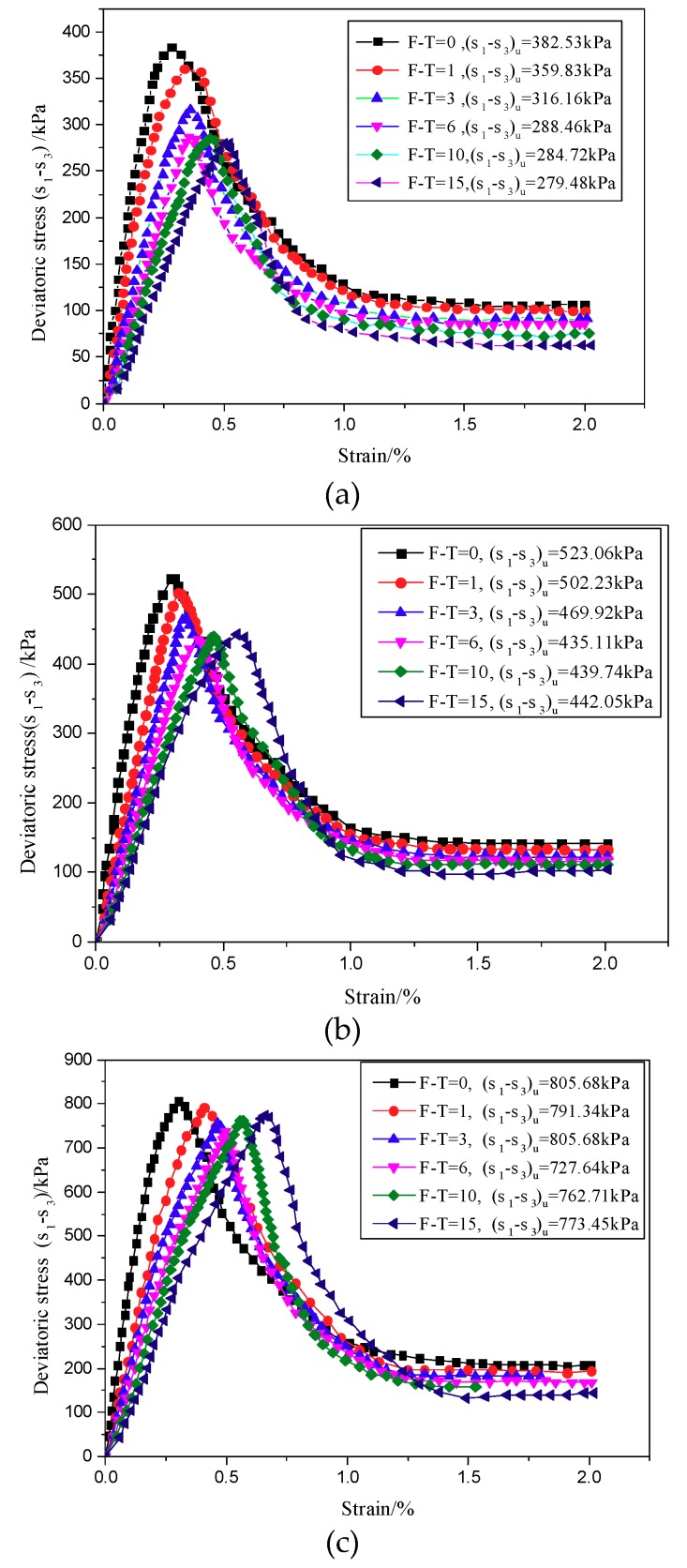
Triaxial shear tests of samples treated with different freeze-thaw cycle numbers. (**a**) σ_3_ = 50 kPa; (**b**) σ_3_ = 100 kPa; (**c**) σ_3_ = 200 kPa.

The stress–strain curves of the CU test under confining pressures of 50, 100, and 200 kPa are shown in Figure 4.

As seen in Figure 4, the stress–strain curves of CIAS exhibit significant peak values, showing obvious strain softening characteristics. The samples reveal brittle shear failure characteristics in the shear test process. The peak strength before freeze-thaw cycling is approximately three times that of the residual strength. Under a confining pressure of 50 kPa, the peak strength of the unfrozen specimens is approximately 382.53 kPa, and the residual strength is 106.55 kPa. After 15 freeze-thaw cycles, the peak strength and residual strength of the specimens decreased to 279.48 and 62.88 kPa, respectively. The first three freeze-thaw cycles have a significant influence on the peak strength, whereas the shear strength remains stable through the subsequent freeze-thaw cycles.

**Figure 5:**
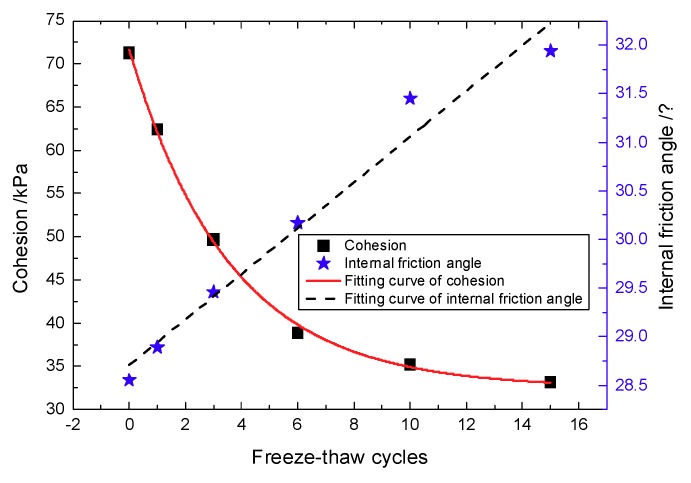
Effects of freeze-thaw cycle numbers on shear strength parameters.

The shear strength parameters c,ϕ correspond to different freeze-thaw cycle numbers and were calculated according to a group of Mohr’s circles with the deviating stresses corresponding to confining pressures of 50, 100, and 200 kPa, respectively. The law of variation of the shear strength parameters with the number of freeze-thaw cycles is shown in Figure 5.

As seen in Figure 5, cohesion decreases with an increasing number of freeze-thaw cycles, whereas the internal friction angle increases with freeze-thaw cycles. This is mainly because the cement products between the aeolian sand particles were destroyed by the frost heave force, causing the particles to denude from the cement, which enhanced the friction between the particles. The shear strength of the specimens is composed of cohesion and internal friction angle. As shown in Figure 4, it can be seen that both the shear strength of the specimens and the number of freeze-thaw cycles first decrease and then increase. This is the result of the combined action of cohesion and internal friction angle.

**Figure 6:**
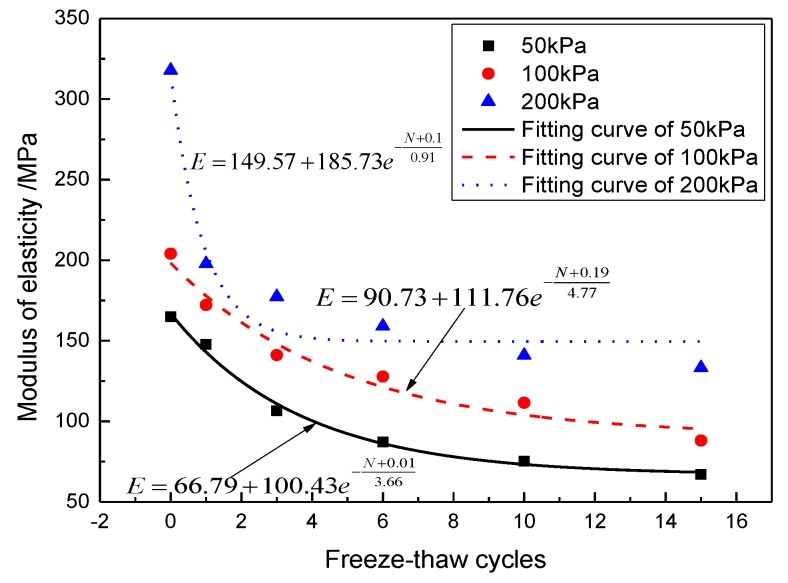
Variation of the modulus of elasticity.

As seen from Figure 4, there was no explicit relationship between peak shear strength and the number of freeze-thaw cycles, although the number of freeze-thaw cycles has a significant effect on the elastic modulus. Therefore, the elastic modulus can be used as an index to measure the effect of freeze-thaw cycle number on the mechanical properties. The strain of 0.25% can be regarded as the end of the linear elastic stage. In the field of damage theory, the "elastic modulus method" is a method based on the assumption of strain equivalence to define the damage variables based on the elastic modulus before and after suffering damage. The attenuation law and fitting equation of the elastic modulus under different confining pressures are shown in Figure 6.

After the first three freeze-thaw cycles, the elastic modulus of the modified aeolian soil dramatically decreases. In Figure 6, the slope of the curve decreases rapidly, accounting for more than 10% of the initial elastic modulus. Under the confining pressure of 200 kPa, the modulus of elasticity decreased by 37.33% after the first freeze-thaw cycle. After three freeze-thaw cycles, the modulus of elasticity remains stable, indicating that the sample is less affected with longer cycle numbers. Under the confining pressures of 100 and 200 kPa, the elastic modulus is higher than that of the sample processed at 50 kPa after three freeze-thaw cycles, indicating that the high confining pressure can restrain the damage caused by the freeze-thaw cycles and that CIAS can maintain good elastic characteristics. Therefore, the initial freeze-thaw cycles have a significant effect on the mechanical properties of the specimens, whereas the effect of the freeze-thaw cycle numbers on the mechanical properties of the CIAS is not significant at long cycle times because a new equilibrium state is achieved.

## 4. CT Scanning of Pore Evolution

### 4.1. CT Scanning Technology

**Figure 7:**
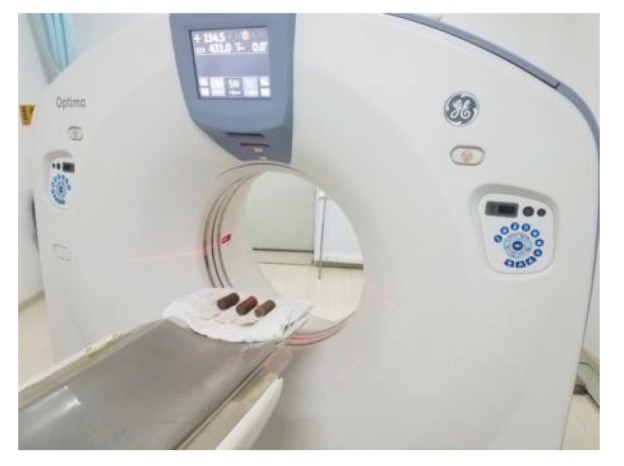
Type GE Optima 520 medical computed tomography (CT) scanner.

**Figure 8:**
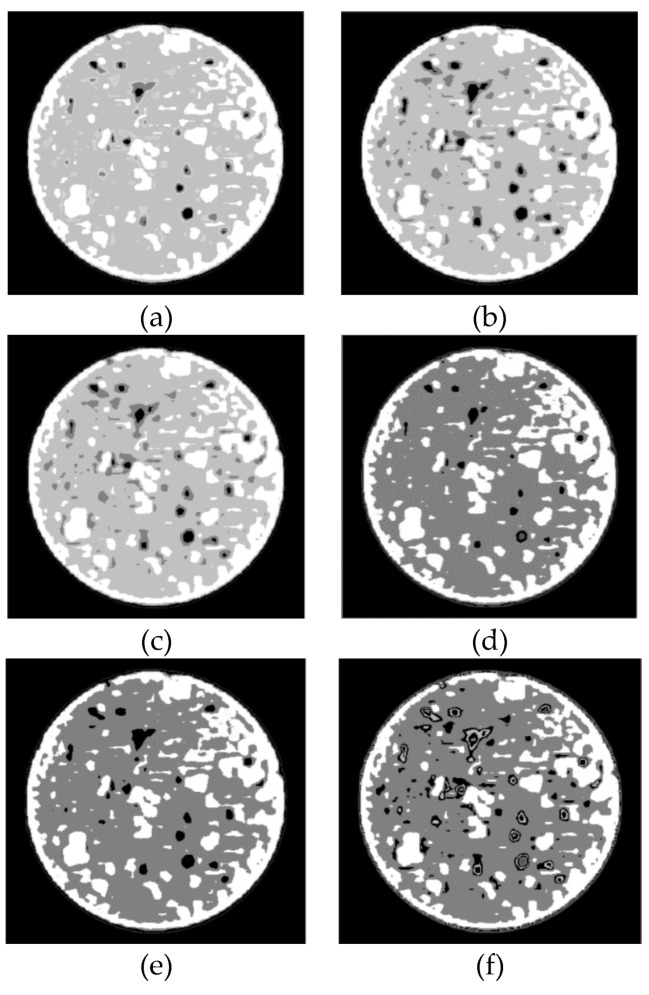
Grayscale images obtained by CT scanning. (**a**) Freeze-thaw = 0; (**b**) Freeze-thaw = 1; (**c**) Freeze-thaw = 3; (**d**) Freeze-thaw = 6; (**e**) Freeze-thaw = 10; (**f**) Freeze-thaw = 15.

The evolution of aeolian sand after freeze-thaw cycling was observed using a GE Optima 520 CT scanner (produced by GE company, Boston, MA, USA ) (Figure 7). The CT value of the CT scanner ranges from −1024 to 3071 Hu, and each scan be completed within 5–10 s. The spatial distribution law is 0.8, the density contrast resolution is 0.5%, and the minimum scanning layer thickness is 0.625 mm. The gray images obtained from CT scanning after 0, 1, 3, 6, 10, and 15 freeze-thaw cycles are shown in Figure 8, where the black regions represent the pore areas, the white regions represent the particle skeleton, and the gray regions represent the excess area between the pores and particles.

**Figure 9:**
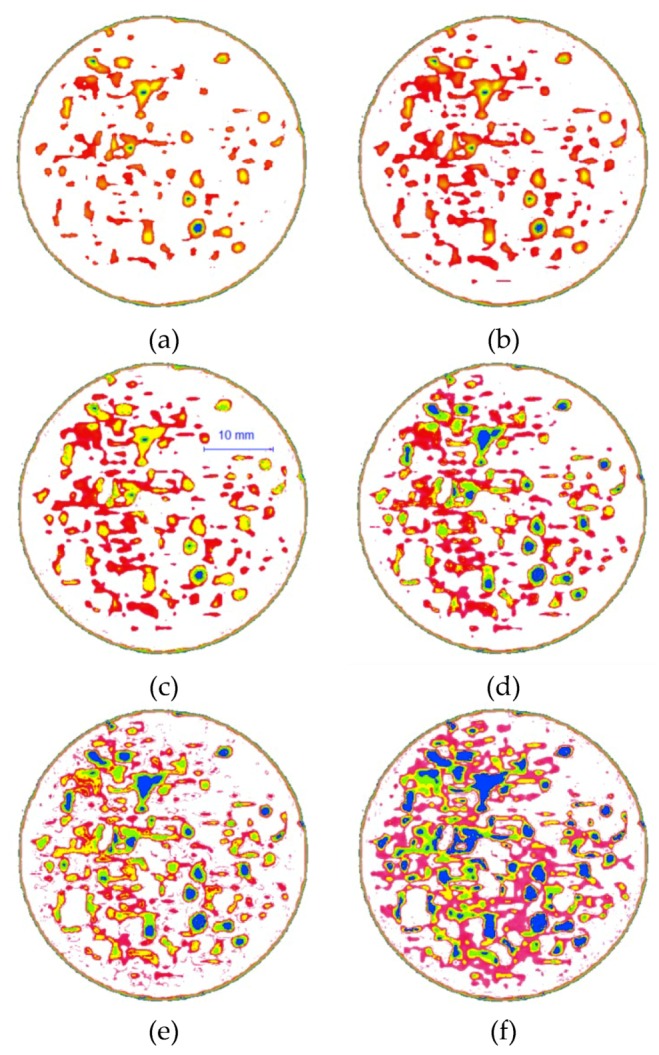
Images processed using the pseudocolor technique. (**a**) Freeze-thaw = 0; (**b**) Freeze-thaw = 1; (**c**) Freeze-thaw = 3; (**d**) Freeze-thaw = 6; (**e**) Freeze-thaw = 10; (**f**) Freeze-thaw = 15.

Based on the differences between the image colors and CT values, the image is divided into four color levels, as listed in Table 4, and the results of the pseudocolor processing and partitioning are shown in Figure 9.

The low-density areas represent regions of weak connection or particles that are entirely unconnected. Hence, the low-density areas along with the porous regions are regarded as uniform damage as a result of the freeze-thaw cycles. Figure 9 represents the area of damage that develops in the CIAS before and after freeze-thaw cycling. The cracking is randomly distributed inside the CIAS, presenting with an irregular shape and obvious self-similar characteristics. As the number of freeze-thaw cycles increases, the low-density area is gradually enlarged, indicating that the original pore features have continuously expanded to form larger cracks, which further increases the degree of irregularity.

The pink area in the sample increases while the white area decreases, indicating that the particle skeleton of CIAS weakens as the density of the sample decreases. Both the blue-green area and yellow area expand continuously. It can be seen that the pink area first gradually turns into the yellow area and then turns into the blue-green area. This is the dynamic evolution process associated with freeze-thaw damage. With the periodic manifestation of the frost-heave force, the porous region continues to expand, indicating that the frost heave force produced by pore waters is the driving force for the observed freeze-thaw damage.

**Figure 10:**
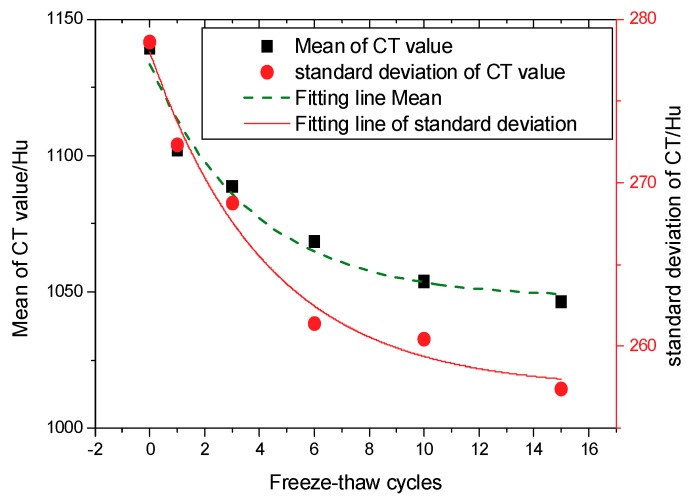
Development of the mean (ME) and standard deviation (SD) of the CT values.

The mean (ME) and standard deviation (SD) of the CT values are two important statistical and quantitative indicators for describing X-ray penetration ability. These values can be used to describe the damage evolution law during freeze-thaw cycling. The laws of variation of ME and SD of the CT values are shown in Figure 10. As seen from Figure 10, both the ME and SD values decrease with increasing freeze-thaw cycle numbers, an observation that is especially significant in the first three freeze-thaw cycles, where the slope changes most rapidly. After three freeze-thaw cycles, the curves tend to stabilize. During the freezing process, the internal water migrates to the outer surface of the sample where it continuously freezes, resulting in the formation of voids within the sample and the expansion of larger cracks. This is why the freeze-thaw damage is most prominent during the initial freeze-thaw process.

To further explore the law of pore evolution after freeze-thaw cycling, the ME and SD curves are fitted in the form of a negative exponential function, as follows.

(1) $$y_{ME}=1047.72+90.01\times e^{-\left(N+0.184\right)/3.72},$$

(2) $$y_{SD}=257.38+20.74\times e^{-\left(N+0.253\right)/4.29},\;$$

where *N* is the number of freeze-thaw cycles, and *e* is the base of natural logarithms.

The correlation coefficients *R*^2^ of the fittings are 0.9223 and 0.9528, respectively, indicating that Equations (1) and (2) can properly describe the changes observed in the ME and SD of the CT values.

### 4.2. Analysis of Light Intensity after Freeze-Thaw Cycling

Light intensity can be used to describe the ability of an X-ray source to penetrate through the interior of the CIAS samples. Areas of low light intensity indicate that X-rays can easily penetrate the samples. The light intensity can be used to characterize the density change law of each position in the CIAS after freeze-thaw cycling; therefore, the law describing the evolution of freeze-thaw damage can reasonably be determined.

**Figure 11:**
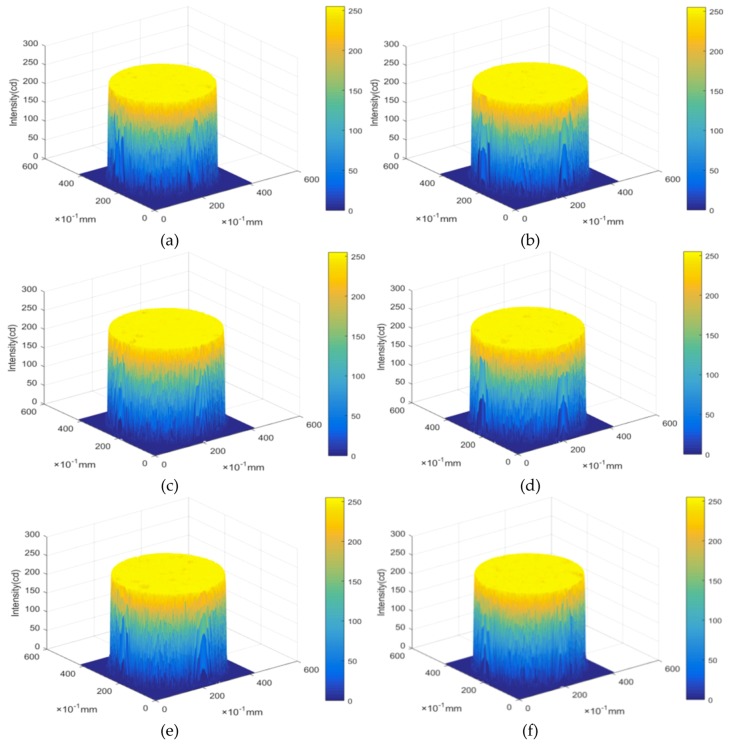
Variation of light intensity after freeze-thaw cycling. (**a**) Freeze-thaw = 0; (**b**) Freeze-thaw = 1; (**c**) Freeze-thaw = 3; (**d**) Freeze-thaw = 6; (**e**) Freeze-thaw = 10; (**f**) Freeze-thaw = 15.

**Figure 12:**
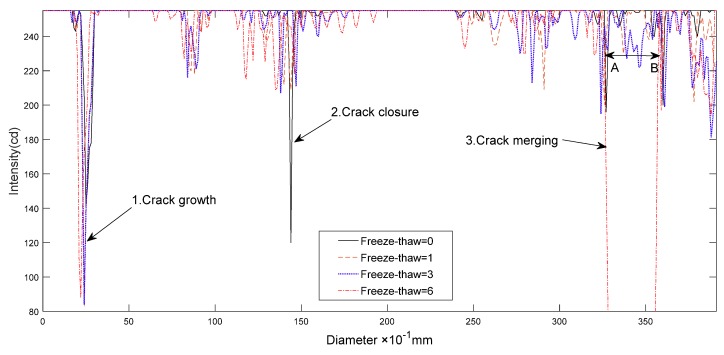
Development trend of the cracks after freeze-thaw cycling.

The light intensity distribution law was developed using MATLAB image processing technology after freeze-thaw cycling of the CIAS samples, as shown in Figure 11, and the development trend followed by the cracks is shown in Figure 12.

As seen in Figure 12, three approximate forms of fracture development were affected by freeze-thaw cycling, as described here. (1) Crack growth: this is the most common process of pore development, occurring in an environment where the pore water is well developed and pore tightness is good. Due to the frost heave force, the small pores merge, growing larger in size, as in case 1 in Figure 12. (2) Crack closure: this situation is typical in an environment where there is less pore water and poor pore tightness. The pore water can easily be drained through pore passages, where many small, closed, and water-rich pores are in close proximity. The frost heaving force promotes pore expansion and compaction of the closed pores, as in case 2 in Figure 12. (3) Crack merging: this phenomenon often occurs where the distribution of microconfined pores is relatively large. Many micropores expand and break through adjacent walls, then merge into larger pores, which could cause extensive damage, as in case 3 in Figure 12.

### 4.3. Trend of Fractal Dimensions of Pore Evolution

**Figure 13:**
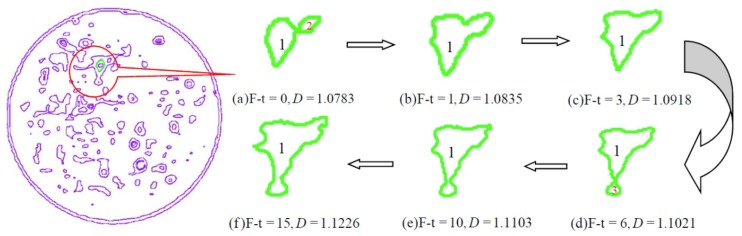
Fractal evolution of the pore margins after freeze-thaw cycling. (**a**) Freeze-thaw = 0; (**b**) Freeze-thaw = 1; (**c**) Freeze-thaw = 3; (**d**) Freeze-thaw = 6; (**e**) Freeze-thaw = 10; (**f**) Freeze-thaw = 15.

As for the disorder associated with pore evolution and the irregular evolutionary characteristics of the pore edge after freeze-thaw cycling, fractal theory, which is a branch of modern mathematics, can be adopted to describe the degree of irregularity of complex graphics. In fractal space, a fractal dimension can be used to describe the complexity of the pore edge. In this paper, the fractal dimension is calculated by using the box covering method in which the size of the square box is continuously reduced until the pore edge can be covered with the same size box, and $L_{1}=a/3$, ${L_{2}=a/3}^{2}$,………, $L_{n}=a/3^{n}$ (where *a* is the initial size of the box and *L_n_* is the size of the *n*^th^ fractal iteration box). The fractal dimension of the pore edge after freeze-thaw cycling can be expressed as Equation (3). Three adjacent pores (pore 1, pore 2, and pore 3) before freeze-thaw cycling are selected for analysis (see Figure 13).

(3) $$D_{N}=\underset{L_{n}\to 0}{\lim}\frac{\log\left[\mathrm{count}\left(L_{n}\right)\right]}{\log L_{n}}$$

where $\mathrm{count}\left(L_{n}\right)$ represents the number of boxes needed to cover the pore edge when the box size is $L_{n}$.

Before freeze-thaw cycling, initial defects are present in the CIAS sample, and trends that pore 1, pore 2, and pore 3 follow as they change over time are shown in Figure 13a–f. As the freeze-thaw cycle number is increased, the area of each pore also increases, the edge of the pore becomes wrinkled and tortuous, and the degree of complexity of the pore edge gradually increases. When the number of freeze-thaw cycles exceeds six, pore 1 and pore 3 make contact and gradually merge into one larger pore. In addition, both the complexity of the pore edge and fractal dimension of the system increase as this cycle number is exceeded.

**Figure 14:**
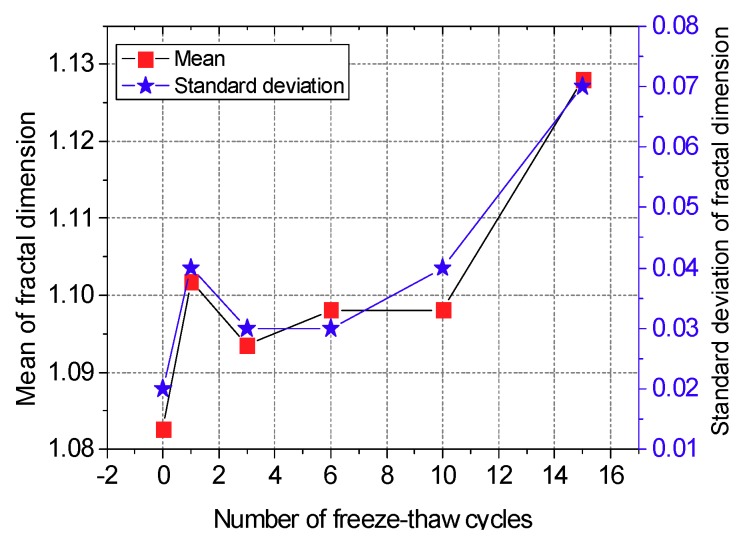
Variation of the statistical parameters of the fractal dimension.

**Figure 15:**
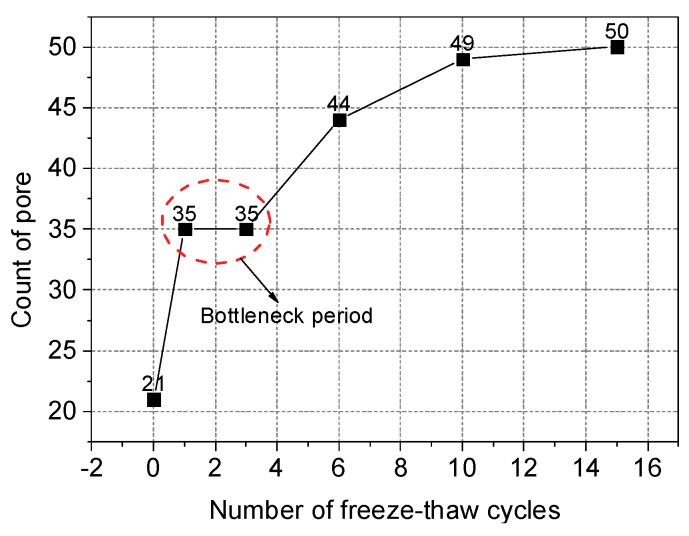
Variation of the number of pores.

The frost heaving force in the newly formed pore is uniform, thus the edge of the pore is relatively smooth and its fractal dimension is relatively low. As the cold peak surface of the water in the pore is random, the pore edge grows increasingly rough. Therefore, the average fractal dimension of the pore edges in the CIAS specimen is determined by the competition between the new and the original pore edge structures. The variations of the ME and SD of the fractal dimension of the pore are shown in Figure 14, and the number of pores is shown in Figure 15.

As seen in Figure 14 and Figure 15, the mean of the fractal dimension fluctuates as the number of freeze-thaw cycles is increased. After the first freeze-thaw cycle, both the fractal dimension and the number of pores dramatically increase, indicating that there are more primary pores at this time. As the frost heaving force causes the most serious damage to the original pores, the irregular degree of the pore edge increases.

After the first freeze-thaw cycle, the number of pores in the sample remains constant, but the fractal dimension decreases, indicating that the rate of combination of original pores and the increase in the number of new pores have reached a dynamic balance. Both the number of pores and the fractal dimension of the system increase after more than three freeze-thaw cycles, indicating that the pore penetration speed is reduced. As a result, the frost heaving force begins to play an important role in expanding the volume of new pores. When the freeze-thaw cycle number is greater than 10, the change in pore number is small, but the fractal dimension increases significantly; this indicates that no primary pores remain at this time. The original pores have been destroyed by the frost heave force, and as a result, the system has entered a freeze-thaw instability stage, with its bearing capacity reaching a minimum.

It can also be seen from Figure 13 that the spacing between pore 1 and pore 2 is small, whereas the spacing between pore 1 and pore 3 is large. The penetration of a given pore begins at the location where the pore’s wall is the weakest, i.e., where the tensile strength of the wall is the lowest. As seen from Figure 13, the number of pores has entered a "bottleneck" stage between the first and third cycle numbers, although the number of pores once more begins to increase after more than three freeze-thaw cycles.

### 4.4. Evolution of Critical Crack Length

**Figure 16:**
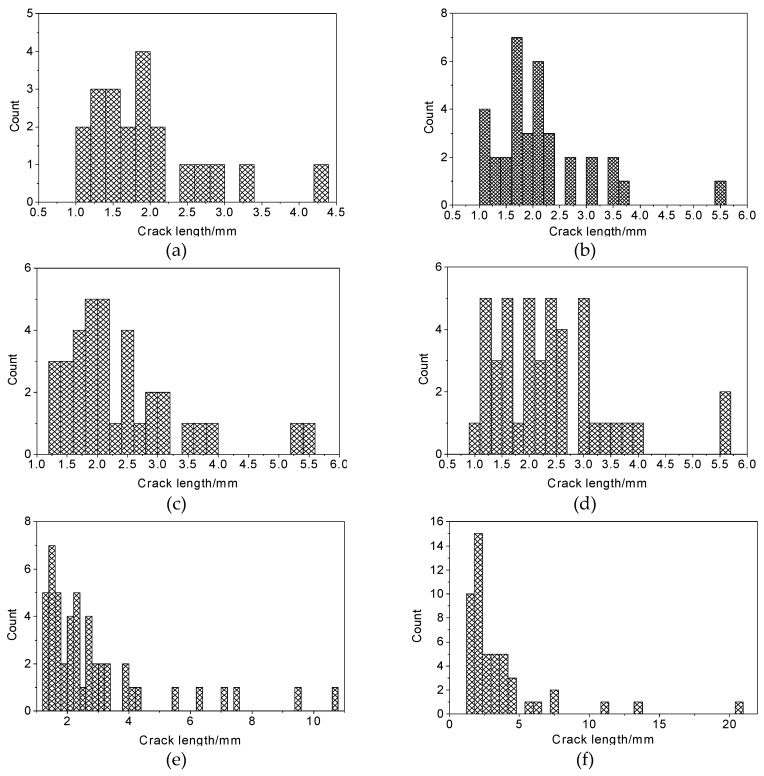
Distribution of crack length for various freeze-thaw cycle numbers. (**a**) Freeze-thaw = 0; (**b**) Freeze-thaw = 1; (**c**) Freeze-thaw = 3; (**d**) Freeze-thaw = 6; (**e**) Freeze-thaw = 10; (**f**) Freeze-thaw = 15.

Critical crack length is an important parameter for describing the damage degree in a CIAS sample. According to our results, fracture length is stable within a certain range of cycles and then grows continuously once a certain number of cycles has been exceeded, until the crack length becomes unstable and the crack is destroyed. The distribution of crack lengths after freeze-thaw cycling is shown in Figure 16.

From Figure 16, it can be seen that the length of a given crack tends to increase after freeze-thaw cycling. The length of the cracks determines the characteristics of damage development in the CIAS due to freeze-thaw cycling. According to the parameter of crack length, three types of cracks are present in the system. If *α* < *α*_cr_, then the crack is stable. This kind of crack imparts little damage to the specimen of CIAS, and as a result, the specimen’s mechanical properties are in general unaffected. If *α* = *α*_cr_, the crack is called a critical crack. This type of crack is in the equilibrium state between the stable stage and unstable stage. If *α* > *α*_cr_, the excess strain energy causes the crack to expand. At this time, the crack is called an unstable crack. This kind of crack causes the greatest damage to the specimen and mechanical properties will be greatly reduced.

**Figure 17:**
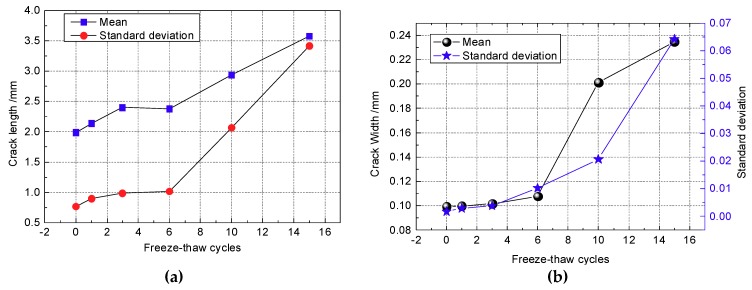
Variation of the statistical parameters of crack length evolution. (**a**) Crack length; (**b**) crack width.

The mean and standard deviation of the crack lengths and crack widths are used to describe the variation of crack length in the CIAS system, as shown in Figure 17, where the trends are plotted against increasing cycle number.

As seen in Figure 17, both the mean and standard deviation of the crack length and width increase with the number of freeze-thaw cycles. The rate of increase of crack length and width in the first six freeze-thaw cycles is relatively low; then, the mean and standard deviation of crack length and width increase rapidly after more than six freeze-thaw cycles. 

Therefore, it is inferred that any unstable cracks within the system develop rapidly after more than six freeze-thaw cycles. Based on Griffith fracture theory, the critical crack half-length is:

(4) $$a_{cr}=\frac{2{\hat{E}}_{N}\gamma }{\pi \sigma_{\Delta T}^{2}},$$

where ${\hat{E}}_{N}$ is the elastic modulus after the *N*^th^ freeze-thaw cycle, *γ* is the Gibbs free energy density of CIAS, and $\sigma_{\Delta T}^{}$ is the maximum thermal stress of ice in the course of freeze-thaw cycling. The critical crack length of CIAS is related to the maximum frost heave force in the freeze-thaw process. The frost heave force can be calculated by Equation (5).

(5) $$\sigma_{\Delta T}^{}=\alpha_{ic}E_{ic}^{}\Delta T,$$

where *α_ic_* is the linear expansion coefficient of ice, and *E_ic_* is the elastic modulus, and ∆*T* is the range of the freeze-thaw temperatures during a cycle. Combining Equation (5) with Equation (4), the relationship of the critical crack half-length and ∆*T* can be construed as follows.

(6) $$a_{cr}=\frac{2{\hat{E}}_{N}\gamma_{cs}}{\pi \alpha_{ic}^{2}E_{ic}^{2}{\left(\Delta T\right)}^{2}}.$$

**Figure 18:**
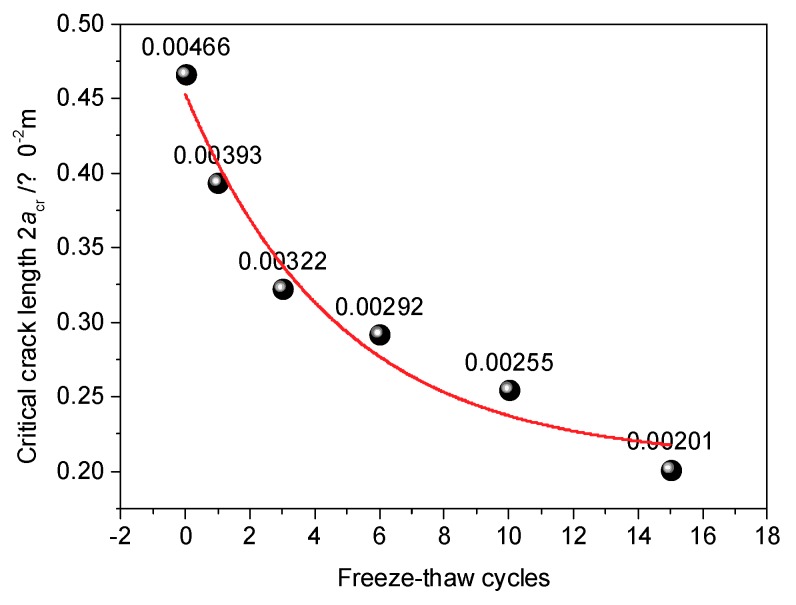
Variation of critical crack length with freeze-thaw cycle number.

The Gibbs free energy density of CIAS can be set as *γ_cs_* = 1.31 × 10^−2^ J/m^2^ for *α_ic_* = 5.1 × 10^−5^ C^−1^, *E_ic_* = 53 MPa and ∆*T* = −10 °C. Figure 6 shows the elastic modulus of CIAS with increasing freeze-thaw cycle number. The variation of critical crack length with freeze-thaw cycle number under the confining pressure of 100 kPa is shown in Figure 18.

The critical length (|AB| in Figure 12) of a crack with increasing freeze-thaw cycle number follows the trend of a negative exponential change, thus the fitting equation is constructed as follows:

(7) $$2a_{cr}=0.00207+0.00255\times \exp\left[-\frac{N+0.1864}{4.77}\right],$$

where *N* is the number of freeze-thaw cycles, and the correlation coefficient *R*^2^ of the fitting curve is 0.9243, indicating that the fitting equation can reasonably describe the trend exhibited by the critical crack length over time.

**Figure 19:**
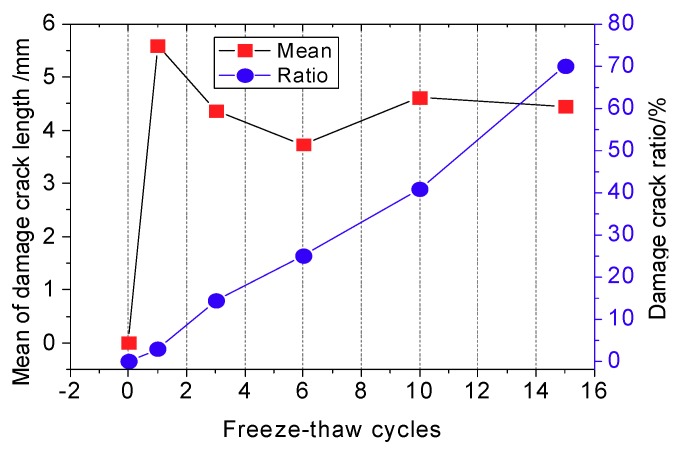
Evolution of damage cracks in the CIAS system.

The critical cracks and unstable cracks are collectively called damage cracks. The mean of the damage crack length and the ratio of damage cracks (ratio of damage cracks = the number of damage cracks/the total count of cracks) are shown in Figure 19.

As seen in Figure 19, the mean of crack length first increases, then decreases and then once more increases with the increase of freeze-thaw cycle number. In contrast, the proportion of damage cracks present in the sample increases continuously with an increasing number of freeze-thaw cycles. After the first freeze-thaw cycle, the average length of a damage crack develops rapidly, mainly due to the expansion of unstable cracks. After more than three freeze-thaw cycles, the mean damage crack length begins to decrease, and the proportion of damage cracks begin to increase, indicating that the original stable cracks have now become critical cracks and unstable cracks. After more than six freeze-thaw cycles, both the critical crack length and the proportion of damage cracks follow increasing trends.

## 5. Evolution of Freeze-Thaw Damage

### 5.1. Statistical Distribution of Pore Area

**Figure 20:**
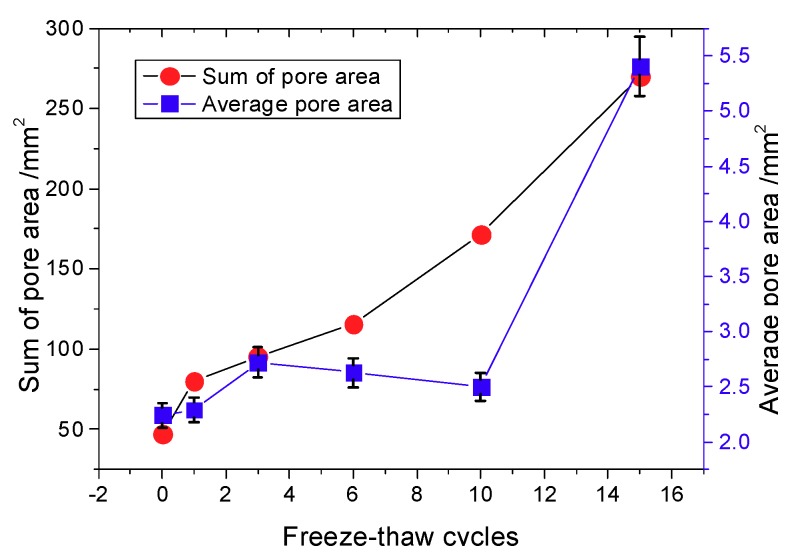
Distribution of pore area with freeze-thaw cycle number.

Due to the random distribution of pore size and the pore evolution process observed during freeze-thaw cycling, a statistical theory was adopted to analyze pore evolution. The variation of the average pore area with the number of freeze-thaw cycles is shown in Figure 20.

**Figure 21:**
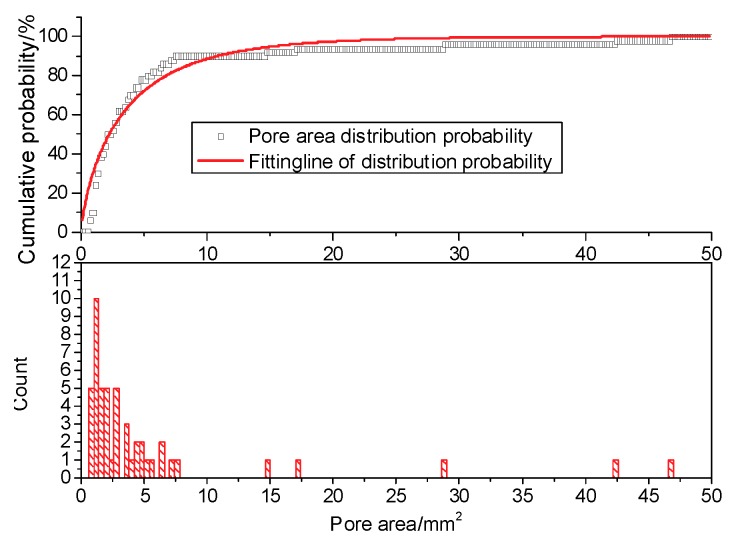
Pore area accumulation probability curve.

The cross-sectional area of a cylindrical specimen is π × (19.55 mm)^2^ = 1200.12 mm^2^. The distribution laws of the cumulative probability and pore count are shown in Figure 21.

As the distribution function curve crosses the origin of the coordinate axis, the Weibull distribution function with two parameters can be adopted to describe the distribution law, as follows [28]:

(8) $$F\left(x\right)=1-\exp\left[-{\left(\frac{x}{F}\right)}^{m}\right],$$

where x is a random statistical variable of pore area, and *m* and *F* are statistical parameters of the Weibull distribution function. When *m* = 1, the system changes into an exponential distribution function. When *m* = 3.6, the system is close to a normal distribution function. When 1 < *m <* 3.6 or *m* > 3.6, the distribution function is deviation front, such that the larger *m* is, the more concentrated the pore area is. The variation of the Weibull distribution parameters *m* and *F* and the correlation coefficient *R*^2^ with the number of freeze-thaw cycles are shown in Table 5.

**Figure 22:**
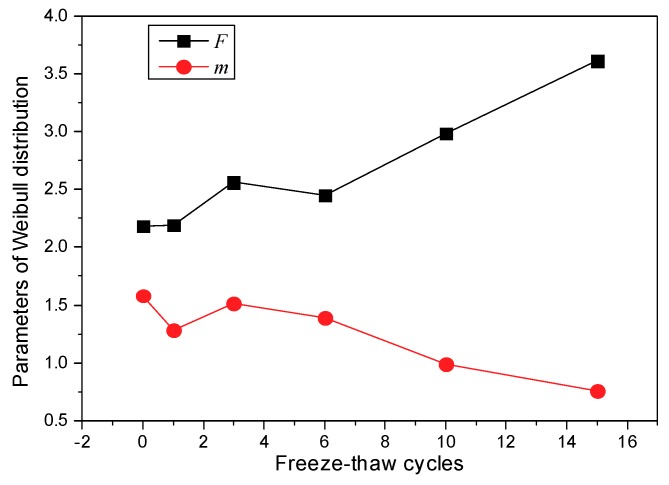
Variation of the Weibull distribution parameters.

The trends followed by parameters *m* and *F* of the Weibull distribution function in Equation (7) after freeze-thaw cycling are shown in Figure 22.

As seen in Figure 22, *m* decreases with increasing freeze-thaw cycle number, whereas *F* increases with increasing freeze-thaw cycle number. When the number of freeze-thaw cycles exceeds 10, the distribution function changes from a frontal distribution to a negative exponential distribution. The distribution characteristic of the pore area before freeze-thaw cycling is relatively uniform. As the number of freeze-thaw cycles increases, the degree of nonuniformity of the pore area increases.

### 5.2. Evolution of Freeze-Thaw Damage

To determine the parameters associated with freeze-thaw damage, the following assumptions are declared:

(a) The freeze-thaw damage is caused by the development of pore expansion due to the frost heaving force;
(b) The density around the pore changes uniformly and continuously; and
(c) The mechanical properties of the undamaged zone are isotropic.

The following are declared: the cross-sectional area of the original sample is $A_{0}$, the pore area after freeze-thaw cycling is $A_{\omega }$, the pore area before freeze-thaw cycling is $A_{\omega 0}$, the pore area after freeze-thaw cycling is $A_{\omega N}$, and the effective bearing area is $A_{\mathrm{ef}}$. Finally, based on these parameters, the continuity factor φ and damage factor $\omega_{N}$ can be respectively defined as [29]:

(9) $$\varphi =\frac{A_{\mathrm{ef}}}{A_{0}},\;\omega_{N}=\frac{A_{\omega N}\;A_{\omega 0}}{A_{0}}.$$

Based on the relationship between total stress σ and effective stress $\sigma_{\mathrm{ef}}$, we obtain:

(10) $$\frac{\sigma }{\sigma_{\mathrm{ef}}}=\frac{A_{\mathrm{ef}}}{A_{0}}=1-\omega_{N}=1-\frac{A_{\omega N}-A_{\omega 0}}{A_{0}}.$$

According to Lemaitre’s strain equivalence principle, the damage constitutive relation of CIAS satisfies [30]:

(11) $$\sigma ={\hat{E}}_{N}\varepsilon ,$$

(12) $$\sigma_{ef}=E_{0}\varepsilon ,\;$$

where $E_{0}$ is the initial nondestructive modulus of elasticity before freeze-thaw cycling, and ${\hat{E}}_{N}$ is the modulus of elasticity after freeze-thaw cycling.

By introducing Equations (11) and (12) into Equation (10), we obtain:

(13) $${\hat{E}}_{N}=E_{0}\left(1-\frac{A_{\omega N}-A_{\omega 0}}{A_{0}}\right).$$

**Figure 23:**
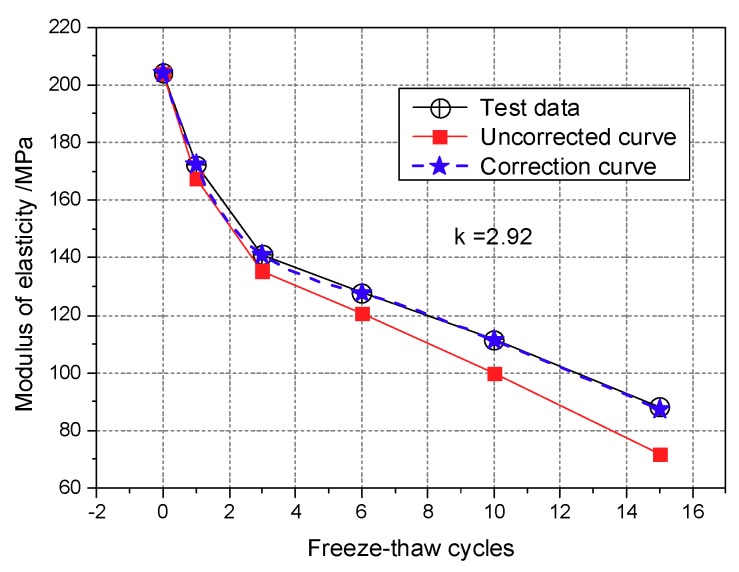
Prediction of the elastic modulus trend due to damage.

The initial elastic modulus under the confining pressure of 100 kPa is 204.01 MPa at the temperature of −10 °C. According to the data presented in Figure 20 and by using Equation (13), we can calculate the damage modulus of elasticity, ${\hat{E}}_{N}$. A comparison between the calculated results and the measured values is shown in Figure 23.

As seen in Figure 14, the calculated values and the measured values deviate, likely due to the random nature of the distribution of pore sizes as measured from different CT observation sections. To describe effectively the triaxial damage based on the CT scanning results, a correction parameter should be introduced to modify Equation (13) as follows:

(14) $${\hat{E}}_{N}=E_{0}\left[1-{\left(\frac{A_{\omega }+A_{\omega 0}}{A_{0}}\right)}^{\kappa }\right],$$

where κ is the modification parameter determined based on the test data and is related to the freezing temperature. The freezing temperature of −10 °C was adopted in this study; hence, the modification parameter κ under the confining pressure of 100 kPa is 2.92. According to the revised calculation results presented in Figure 13, this factor is very close to the experimental results. Equation (14) establishes the mathematical relationship between the elastic modulus and pore area. Considering the effect that freeze-thaw cycling has on improving the mechanical properties of CIAS, the proposed freeze-thaw damage model expressed in Equation (14) is reasonable.

## 6. Conclusions

In this paper, the influence of freeze-thaw cycling on shear strength parameters was determined by using triaxial shear tests. The pore change law during freeze-thaw cycling was obtained from CT scan images. IPP and MATLAB image processing technology were used to process and analyze the CT scan images. The variation trends of the pore area, crack length and fractal dimensions of the pore edges were studied. As a result, the following conclusions are drawn:

(1) Cohesion decreases with the increase of freeze-thaw cycle number, whereas the angle of internal friction increases with the increase of freeze-thaw cycle number. Under the confining pressure of 50 kPa, the shear strength decreases with the increase of freeze-thaw cycle number. In contrast, under the confining pressures of 100 and 200 kPa, the shear strength first decreases and then increases with the increase of freeze-thaw cycle number. The modulus of elasticity decreases with the increase of freeze-thaw cycle number at all of the three confining pressures studied here and follows the negative exponent attenuation law.
(2) The range of low-density area in the CT scan images continuously expands during the freeze-thaw cycling process, and the expansion process is associated with a fractal characteristic. Specifically, the mean fractal dimension of the pore edge is between 1.08 and 1.13, and the standard deviation of fractal dimension of the pore edge is between 0.01 and 0.08.
(3) The critical length of unstable cracks decreases gradually with the increase of freeze-thaw cycle number and follows the attenuation law of a negative exponential function. The coefficient of determination is up to 0.9243. The average length of damaged cracks increases predominantly after the first freeze-thaw cycle and then fluctuates during subsequent freeze-thaw cycles.
(4) The total pore area increases with the increase of freeze-thaw cycle number, and the degree of freeze-thaw damage accumulates during this time. During freeze-thaw cycling, the occurrence of three distinct growth modes leads to the fluctuation of the mean pore area. The damage mechanics model reasonably describes the attenuation law of the elastic modulus after freeze-thaw cycling.

## Figures and Tables

**Table 1:** Basic physical and mechanical parameters of the aeolian sand samples.

Moisture Content (%)	Gravity (kN·m^−3^)	Liquid Limit (%)	Plastic Limit (%)	Maximum Dry Weight (kN·m^−3^)	Compressive Modulus (MPa)	Cohesive Force (kPa)	Internal Friction Angle (°)
21.2	20.1	48.3	22.6	18.1	19.8	0.2	31.4

**Table 2:** Basic physical and mechanical parameters of the aeolian sand samples.

Chemical Composition	Na_2_O	MgO	Al_2_O_3_	SiO_2_	CaO	K_2_O	Others
Content/%	2.16	1.34	24.45	63.5	3.51	2.09	2.95

**Table 3:** Changes of the physical characteristics due to freeze-thaw cycles.

Freeze-Thaw Cycles	Height/mm	Volume/mm^3^	Mass/g
0	80.00	96,009.27	182.41
1	80.36	96,030.12	179.85
3	80.74	96,060.85	177.59
6	80.89	96,084.21	176.76
10	80.98	96,093.65	176.51
15	81.02	96,102.31	175.81

**Table 4:** CT value, intensity, and corresponding substances.

Color	Intensity	CT Value (Hu)	Medium
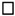	High	1400–2500	Particle skeleton
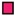	Relatively high	700–1400	Transitional area
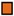	Medium	0–700	Pore water
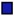 and 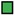	Low	−700–0	Pore

**Table 5:** Variation of Weibull Distribution Parameters.

Freeze-Thaw Cycles	*m*	*F*	*R* ^2^
0	2.18	1.58	0.9874
1	2.19	1.28	0.9817
3	2.56	1.51	0.9817
6	2.45	1.39	0.9839
10	2.98	0.99	0.9597
15	3.61	0.76	0.9326
